# Moonlighting in Mitosis: Analysis of the Mitotic Functions of Transcription and Splicing Factors

**DOI:** 10.3390/cells9061554

**Published:** 2020-06-26

**Authors:** Maria Patrizia Somma, Evgeniya N. Andreyeva, Gera A. Pavlova, Claudia Pellacani, Elisabetta Bucciarelli, Julia V. Popova, Silvia Bonaccorsi, Alexey V. Pindyurin, Maurizio Gatti

**Affiliations:** 1 IBPM CNR c/o Department of Biology and Biotechnology, Sapienza University of Rome, 00185 Rome, Italy; patrizia.somma@uniroma1.it (M.P.S.); claudia.pellacani@uniroma1.it (C.P.); elisabetta.bucciarelli@uniroma1.it (E.B.); 2 Institute of Molecular and Cellular Biology, Siberian Branch of RAS, 630090 Novosibirsk, Russia; andreeva@mcb.nsc.ru (E.N.A.); gpavlova@exseed.ed.ac.uk (G.A.P.); popova@mcb.nsc.ru (J.V.P.); 3 Department of Cell Biology, Albert Einstein College of Medicine, Bronx, New York, NY 10461, USA; 4 Wellcome Centre for Cell Biology, School of Biological Sciences, University of Edinburgh, Edinburgh EH9 3BF, UK; 5 Department of Biology and Biotechnology, Sapienza University of Rome, 00185 Rome, Italy; silvia.bonaccorsi@uniroma1.it

**Keywords:** transcription factors, splicing factors, multifunctional proteins, protein moonlighting, mitosis, microtubules, spindle, centrosomes, kinetochores, midbody

## Abstract

Moonlighting proteins can perform one or more additional functions besides their primary role. It has been posited that a protein can acquire a moonlighting function through a gradual evolutionary process, which is favored when the primary and secondary functions are exerted in different cellular compartments. Transcription factors (TFs) and splicing factors (SFs) control processes that occur in interphase nuclei and are strongly reduced during cell division, and are therefore in a favorable situation to evolve moonlighting mitotic functions. However, recently published moonlighting protein databases, which comprise almost 400 proteins, do not include TFs and SFs with secondary mitotic functions. We searched the literature and found several TFs and SFs with bona fide moonlighting mitotic functions, namely they localize to specific mitotic structure(s), interact with proteins enriched in the same structure(s), and are required for proper morphology and functioning of the structure(s). In addition, we describe TFs and SFs that localize to mitotic structures but cannot be classified as moonlighting proteins due to insufficient data on their biochemical interactions and mitotic roles. Nevertheless, we hypothesize that most TFs and SFs with specific mitotic localizations have either minor or redundant moonlighting functions, or are evolving towards the acquisition of these functions.

## 1. Introduction

In 1999, Constance J. Jeffrey, coined the term “moonlighting protein” to describe a protein that performs one or more additional functions besides its canonical one [1]. Moonlighting proteins play roles in many different biological processes. Recently published manually curated databases (MoonProt 2.0 and MoonDB 2.0) describe almost 400 moonlighting proteins [2,3] and new bifunctional and multifunctional proteins are continuously discovered.

The coexistence of more than one activity in a single protein is the likely result of a gradual evolutionary transition from the original function to a novel function. It has been further posited that protein moonlighting is favored when the novel and the canonical function are exerted in different cellular compartments, and/or in combination with different binding partners, so as to avoid functional conflicts [1,4,5]. Under this view, mitosis seems to be an ideal process to favor acquisition of moonlighting functions, as it is characterized by a dramatic rearrangement of cell structures, accompanied by re-localization and compartmentalization of many proteins. For example, during chromosome condensation, many chromatin proteins are released in the cytoplasm and re-associate with the chromatin only when chromosomes decondense during telophase. Some of these proteins have acquired moonlighting mitotic functions [6,7]. The ISWI and CHD4 chromatin remodeling factors bind the spindle microtubules (MTs) in both *Drosophila* S2 cells and *Xenopus* cells and egg extracts, and are required for spindle assembly and functioning [8,9]. Similarly, the human INO80 ATPase, involved in both DNA replication and chromatin remodeling, binds the mitotic spindle and is required for chromosome segregation [10,11]. Thus, the mitotic spindle can bind and compartmentalize proteins that during interphase are associated with the chromatin and reside within the nucleus.

During mitosis, several cellular processes such as transcription and splicing are strongly reduced [12,13,14], and therefore the proteins involved in these processes are in a very favorable situation to evolve secondary mitotic functions. Indeed, several genome-wide screens carried out in *Drosophila* and human cells have shown that RNAi-mediated depletion of many different transcription factors (TFs) and splicing factors (SFs) results in a variety of mitotic defects [15,16,17,18,19,20] (see Tables S1 and S2). Other TFs and SFs required for mitotic division have been identified in studies on specific proteins or protein complexes (see Table 1 and Table 2 and text below). However, in most cases, it is unclear whether the TF- or SF-dependent mitotic phenotype is due to a direct (moonlighting) function of the factor, or to an indirect effect on transcription or splicing of one or more mRNAs encoding mitotic proteins (henceforth, mitotic mRNAs).

In this review, we analyze the literature on the mitotic roles of TFs and SFs. We include in our TF category not only the canonical DNA binding factors, but also the proteins that regulate transcription without binding to DNA (e.g., co-activators and co-repressors) [21]. Similarly, we consider as SFs both the proteins complexed with small nuclear RNAs (snRNAs) and the proteins that do not directly interact with these RNAs but are required for splicing [22]. We report many examples of TFs and SFs that in addition to their canonical function in transcription and splicing, play moonlighting mitotic roles, as well as cases where the TFs and SFs appear to have indirect mitotic effects controlling the biogenesis of mitotic mRNAs. Defining whether a factor associated with a loss-of-function mitotic phenotype has a direct mitotic role is not straightforward. We classify a TF/SF as a moonlighting mitotic protein when it meets at least three conditions: (i) it localizes to a specific mitotic structure, (ii) biochemically interacts with proteins enriched in the same structure, and (iii) causes defects in this structure upon depletion. Conversely, a TF/SF is classified as an indirect mitotic player when it does not exhibit a mitotic localization and there is biochemical evidence that its loss impairs transcription or splicing of genes encoding mitotic proteins specifically related to the affected mitotic structure. However, for many TFs/SFs that localize to mitotic structures, published information is not sufficient to decide whether they play a direct function during cell division. For example, for some of these factors, there is no information on their biochemical interactions or loss-of-function mitotic phenotype, while knockdown of other factors does not appear to elicit a clear mitotic phenotype. Thus, we classify this heterogeneous group of TFs/SFs as factors with a “putatively direct” mitotic function (Table 1 and Table 2), whose precise assessment requires further investigation.

Overall, we provide a large but non-exhaustive list of TFs and SFs with mitotic functions and critically analyze their mitotic roles. We would like to point out that none of the TFs and SFs with direct mitotic roles (see Table 1 and Table 2 below) are included in the extant moonlighting protein databases [2,3].

## 2. Transcription Factors (TFs) with Mitotic Functions

### 2.1. TFs That Control Mitosis by Regulating the Expression of Mitotic Genes

Several TFs are thought to affect mitosis indirectly through the regulation of transcription or stability of mitotic mRNAs. Forkhead transcription factors (FKH-TFs) have been implicated in the regulation of mitotic gene transcription in both yeast and humans [23,24]. In humans, FKH-TFs (e.g., FOXO3) regulate the expression of mitotic genes such as *cyclin B1* (*CCNB1*) and reductions in the activities of FKH-TFs result in delayed M-G1 transition and defective cytokinesis [23]. In *S. pombe*, most of the mitotic gene promoters targeted by FKH-TFs (e.g., Fkh2) are additionally associated with Sak1, a TF of the RFX family that also appears to control mitosis indirectly [24] (Table 1).

Another TF that is thought to have an indirect mitotic function is Zas1 of *S. pombe*, which controls the expression of a specific set of genes involved in cell division, including the gene encoding the Cnd1 subunit of the condensin complex [25]. Mutations in *Zas1* cause defects in chromosome condensation and segregation, but these defects are not fully rescued by expression of the Cnd1 protein. This finding has led to the suggestion that the mitotic phenotype elicited by Zas1 deficiency might be the consequence of the simultaneous deregulation of multiple mitotic genes [25]. However, the observation that Zas1 remains associated with the chromosomes throughout mitosis, and that this association is essential for its mitotic function [25], does not exclude the possibility that Zas1 directly participates in the chromosome condensation process.

A peculiar form of indirect mitotic regulation is exerted by the ERG transcription factor of the ETS family. In addition to the nucleus, ERG accumulates in cytoplasmic messenger ribonucleoprotein (mRNP) aggregates called processing bodies (PBs), which contain the RNA degradation machinery. Consistent with this localization, ERG promotes mRNA decay by binding the CCR4–NOT deadenylation complex. ERG depletion in HeLa cells results in impaired degradation of a subset of mRNAs including those encoding the Aurora kinase A (AURKA) and Aurora kinase B (AURKB). Accumulation of these kinases deregulates Aurora signaling, resulting in a variety of mitotic defects, including centrosome fragmentation, morphologically abnormal bipolar spindles and multipolar spindles [26].

### 2.2. TFs That Localize to Centrosomes

**Figure 1:**
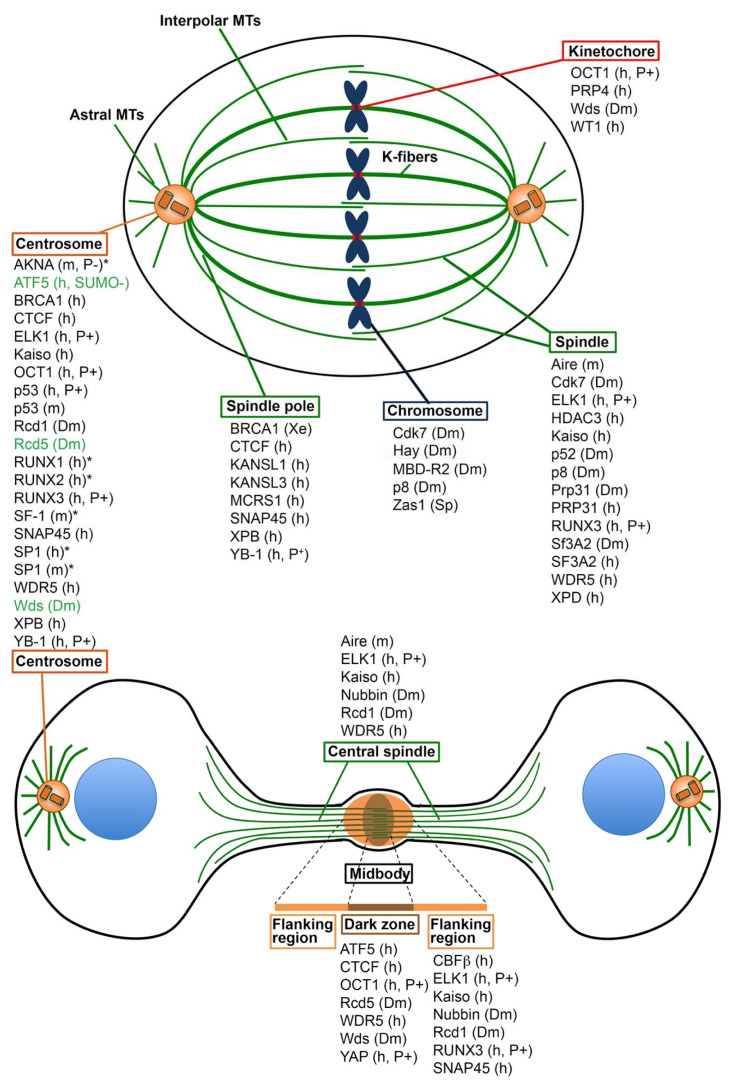
Localization of transcription and splicing factors to mitotic structures of metaphase (top) and late telophase (bottom) cells. Data are from human (h), mouse (m), *Drosophila* (Dm), and *S. pombe* (Sp) cells and from spindles assembled in *Xenopus* egg extracts (Xe). Protein localization in the schematic metaphase is also observed in prometaphases and anaphases. Likewise, the protein localization pattern in the schematic late telophase is comparable to that seen in earlier stages of telophase. Of the proteins that localize to the centrosomes, most are present from prophase to telophase, but some (depicted in green) are not detected in telophase centrosomes. For other centrosomal proteins (marked by an asterisk), lack of pertinent images did not permit an assessment of their presence in telophase centrosomes. The midbody at the center of the cellular bridge consists of a dark zone that contains interdigitating antiparallel MTs associated with several proteins, including centralspindlin (MKLP1/KIF23 and CYK4/RACGAP1), CIT-K, KIF14, and anillin. These and other proteins form a dense cluster (midbody ring) that impedes access of anti-tubulin antibodies, resulting in a dark zone after immunostaining. The flanking regions also contain several proteins, including those of the ESCRT machinery that mediates abscission, which can occur at either side of the dark zone. Localization of some proteins is phosphorylation (P^+^), dephosphorylation (P^−^), or desumoylation (SUMO^−^) dependent.

There are many TFs that accumulate at the centrosomes (Table 1; Figure 1). The centrosomes are self-assembling organelles that nucleate the spindle MTs during cell division. They are comprised of a pair of orthogonally arranged rod-like centrioles surrounded by pericentriolar material (PCM), which contains several proteins necessary for MT nucleation, including γ-tubulin (TUBG1) [27,28]. In mammals, a centriole pair consists of a mother centriole characterized by the presence of subdistal appendages, and a younger daughter centriole that grows from the mother during interphase; the two centrioles remain attached to each other until anaphase, when they are disengaged by separase [27]. Some TFs meet our three criteria for a moonlighting function at the centrosomes, namely they localize to the centrosomes, physically interact with centrosomal proteins and are required for centrosome structure, duplication, or MT nucleation activity. Other TFs meet only one or two of these three criteria, and it is therefore currently unclear whether they play direct functions at the centrosomes.

An example of a moonlighting TF with a centrosomal function is the well-known tumor suppressor BRCA1. BRCA1 is a multifunctional protein that is included in several different complexes primarily involved in DNA replication and repair. BRCA1 can also associate with the RNA polymerase II holoenzyme and some well-known TFs (TFIIF/GTF2F1, TFIIE/GTF2E1, and TFIIH/GTF2H1; here and henceforth, the commonly used abbreviation for a human protein and its HUGO-based symbol are separated by a slash), acting as a transcriptional regulator [29,30]. In addition, BRCA1 is enriched at the centrosomes where it interacts with the BRCA1-associated RING domain protein (BARD1) and the Obg-like ATPase 1 (OLA1) to form a complex with E3 ligase activity that binds and ubiquitinates γ-tubulin [31,32,33,34,35]. *BRCA1* downregulation causes centrosome amplification and fragmentation in cell lines from mammary tissues [36,37] and in mouse embryonic fibroblast cells [38]. The localization of BRCA1 at the centrosome, its biochemical interaction with γ-tubulin, and the centrosomal phenotype elicited by its deficiency strongly suggest that BRCA1 is directly involved in centrosome maintenance.

The evolutionarily conserved BRCA1 protein is also required for spindle formation induced by sperm nuclei in cytoplasmic extracts from *Xenopus laevis* eggs. In this system [39], BRCA1 is slightly enriched at the spindle poles and physically interacts with BARD1 and the spindle-associated proteins TPX2, NuMa, and XRHAMM. BRCA1 immunodepletion results in morphologically aberrant spindles with unfocused poles and a higher MT density compared to spindles formed in BRCA1-nondepleted extracts [40]. Because *Xenopus* egg extracts can support mitosis independently of transcription and translation, requiring only synthesis of cyclin B [41,42], these findings strongly suggest that BRCA1 directly contributes to centrosome-independent spindle assembly in this in vitro system.

Another remarkable example of a TF with a moonlighting role in centrosome assembly and function is ATF5, a member of the ATF/CREB family of TFs [43]. In human cells, ATF5 accumulates at the centrosomes from G1 through anaphase and dissociates from the centrosomes during late telophase. Consistent with its subcellular localization, ATF5 interacts with multiple centrosomal proteins, including γ-tubulin and pericentrin (PCNT), and is specifically enriched in a cylindrical structure that encircles the proximal end of the mother centriole [44]. ATF5 association with the centrosomes is regulated by SUMOylation; SUMOylated ATF5 does not localize to centrosomes and does not interact with centrosomal proteins [45]. RNAi-mediated downregulation of ATF5 results in impaired centrosomal accumulation of PCM components such as γ-tubulin and PCNT, centriolar fragmentation and formation of multipolar spindles [44]. Interestingly, during late telophase, ATF5 dissociates from centrosomes and accumulates in the midbody [44], the structure in the middle of the bridge that connects the daughter cells during completion of cytokinesis (Figure 1). However, the ATF5 functional role at the midbody, if any, is currently unknown. The midbody forms when the overlapping plus ends of the central spindle MTs are compacted by cytokinetic ring constriction, so as to form a MT-dense region, called dark zone, associated with multiple proteins that impede staining with anti-tubulin antibodies [46,47]. The midbody has a defined protein composition and substructure [47,48], but here we will only refer to the dark zone (DZ) and its flanking regions (FR), because these are the only regions we can appreciate in published figures of late telophases stained for both tubulin and the TF or SF under study (Figure 1).

Another integral centriolar component is AKNA, an AT-hook TF that is required for neurogenesis in mouse brain [49]. Like ATF5, AKNA localizes to the mother centriole, where it is mostly enriched at the subdistal appendages. In contrast with the ATF5 behavior, AKNA dissociates from centrosomes during mitosis upon increased phosphorylation and re-associates with the centrosomes during late telophase/early G1. AKNA recruits and physically interacts with components of the γ-tubulin ring complex (γTuRC), and MT binding proteins such as EB1, P150-Glued (DCTN1), and dynein. RNAi-mediated *AKNA* knockdown results in a significant reduction in centrosome-driven MT regrowth after MT depolymerization with nocodazole, but it is unclear whether this defect underlies a gross alteration in normal spindle assembly [49].

Additional TFs that are likely to play moonlighting functions at the centrosomes are the Y-box binding protein-1 (YB-1/YBX1) and OCT1/POU2F1. YB-1, which is overexpressed in many cancers, has roles in transcription, translation, and splicing, localizes to the centrosomes of human cells in a phosphorylation-dependent manner, and physically interacts with γ-tubulin and PCNT. In YB-1-depleted cells, centrosomes are structurally abnormal and have reduced MT nucleation ability [50,51]. OCT1 is a well-known TF that regulates metabolism and tumorigenicity in humans and mice. Upon phosphorylation in its DNA binding domain, OCT1 localizes to the centrosomes, the central spindle and the midbody dark zone, and to a lesser extent to the kinetochores ([52] and references therein). OCT1 interacts with proteins that are also found in the centrosomes such as BRCA1 [53,54] and PARP-1 [55]. RNAi-mediated OCT1 depletion in HeLa cells results in abnormal mitosis. Similarly, immunodepletion of the OCT1 ortholog from *Xenopus* egg extracts causes defects in spindle assembly, a finding that provides further support for a moonlighting mitotic function of OCT1 [52]. The OCT1 *Drosophila* ortholog, Nubbin, does not appear to localize to centrosomes but is enriched at both the central spindle and the midbody flanking regions [56] (Figure 1). It is currently unknown whether OCT1 and Nubbin have roles in cytokinesis [52,56].

There are several additional TFs that localize to the centrosomes, but it is currently unclear whether they play direct functions in these structures. One of these TFs is the intensively studied p53/TP53 protein that plays crucial roles in the maintenance of genome stability and tumor suppression [57]. p53 controls transcription of genes involved in a variety of cellular processes, including cell cycle, DNA repair, senescence, apoptosis, and autophagy, but also has direct cytoplasmic roles in mitochondrial membrane permeabilization, apoptosis, and possibly autophagy inhibition [57]. p53 localizes to the centrosomes in a variety of human, chicken, and rodent cells [58,59,60]; in human cells, centrosomal localization of p53 is phosphorylation-dependent [61]. Loss of p53 results in centrosome amplification in both human and mouse cells [62,63,64,65,66]. Interestingly, recent work has shown that p53 depletion in human non-transformed cells results in centrosome fragmentation [67]. However, centrosome fragmentation was not observed in p53-deficient mouse cells and human cancer cells, which instead showed centrosome amplification [67], as previously described [65]. Although it has been suggested that p53 might directly contribute to prevention of centrosome amplification [64,65] or fragmentation [67], a compelling evidence for a moonlighting role of p53 at the centrosome is still lacking.

Other TFs that accumulate at the centrosomes of mammalian cells, but do not fit our three criteria for a moonlighting centrosomal function are SP1, SF-1/NR5A1, Kaiso/ZBTB33, CTCF, SNAP45/SNAPC2, and ELK1. SP1, which has been implicated in tumorigenesis, localizes to the centrosomes of mouse and human cells and its loss causes centrosome amplification and decreased centrosome-driven MT nucleation [68]. It has been suggested that SP1 deficiency results in the activation of TORC1 signaling, which leads to centrosome amplification [68]. SF-1 prevents centrosome overduplication in mouse cells; SF-1 depletion leads to an aberrant centrosomal accumulation and activation of the DNA-dependent protein kinase catalytic subunit (DNA-PKcs), which is thought to activate the AKT-dependent signaling cascade that leads to centrosome amplification [69,70]. Kaiso is a BTB/POZ zinc finger protein that acts as a transcriptional repressor; in human cells, it localizes to the centrosomes, the spindle, the central spindle, and the midbody flanking regions (Figure 1), and interacts with both γ-tubulin and PCNT. However, loss of Kaiso does not cause obvious centrosome or mitotic defects [71,72]. CTCF is a transcriptional regulator that physically interacts with Kaiso [73]; it localizes to the centrosomes, the spindle poles and the midbody dark zone in HeLa cells (Figure 1), but the mitotic effects of its depletion were not investigated [74]. SNAP45, a TF required for transcription of snRNA genes, localizes to the centrosomes, the spindle poles, and the midbody flanking regions (Figure 1). In HeLa cells, RNAi-mediated depletion of SNAP45 leads to multiple mitotic defects, including morphologically abnormal spindles and defective chromosome segregation. These aberrant mitotic phenotypes are at least in part the consequence of additional primary defects in chromosome condensation and sister chromatid pairing at metaphase [75]. Phosphorylated ELK1 localizes to the centrosomes, the spindle, the central spindle, and the midbody flanking regions in human cells [76] (Figure 1). ELK1 physically interacts with Aurora A [76], and its loss causes mitotic delay but no obvious mitotic defects [20].

The Runt-related TFs (RUNX1, RUNX2, and RUNX3) are important developmental regulators with roles in carcinogenesis [77]. They also have multiple mitotic roles, but it is currently unclear whether some of these roles are direct. Human RUNX1 positively regulates transcription of genes encoding components of the spindle assembly checkpoint (SAC) machinery such as BUBR1/BUB1B, BUB1, and NEK6 [78]. During human cell mitosis, following RUNX3 phosphorylation, the RUNX proteins localize to the centrosomes and the midbody flanking regions [79] (see Figure 1 for mitotic localization of individual RUNX proteins). It has been also shown that the RUNX factors co-precipitate with centrosomal proteins such as γ-tubulin and the centriolar component rootletin (CROCC) [80]. However, individual depletion of each of the RUNX proteins results in reduced cyclin B1 accumulation and delayed mitotic entry, but does not cause specific centrosome-related mitotic defects [79,80].

Several proteomic studies have shown that the centrosomes contain hundreds of different proteins, but only a fraction of them (approximately one fourth) is known to be involved in centriole structure and duplication or PCM-driven MT nucleation [81,82,83]. The functions of the centrosome-associated proteins that do not appear to be involved in centrosome activity, such as p53, SP1, SF-1, Kaiso, CTCF, ELK1, and RUNX TFs, are unclear. It cannot be excluded that some of these proteins play an as yet undefined direct function required for proper centrosome behavior. Alternatively, it is possible that their localization and accumulation in the centrosome helps them to interact with other proteins hosted in this subcellular structure, or to keep them sequestered until they become necessary for certain cellular processes. Recent work has provided some clues on how proteins without direct centrosome-specific functions can concentrate in this organelle. It has been suggested that centrosome assembly is facilitated by the ability of several centrosomal proteins to undergo liquid–liquid phase transition and form liquid-like droplets that concentrate cytoplasmic components and then fuse to form the PCM around the centrioles [84]. These droplets may include cytoplasmic proteins that will become spurious centrosome components.

Interestingly, the centrosomes harbor several proteins involved in the DNA damage response (DDR), including the ATM, ATR, CHK1, and CHK2 kinases [85]. The ATM and ATR kinases are recruited to the sites of DNA damage and initiate a signaling cascade by phosphorylating the effector CHK1 and CHK2 kinases, ultimately leading to p53 activation [57,85]. There is evidence that DNA damage causes aberrant centriole disengagement, leading to centrosome amplification ([85] and references therein), a condition that is thought to promote tumor initiation and progression [86]. However, it remains unclear whether the centrosomal fractions of the DDR kinases and p53 have roles in DNA damage signaling and/or centrosome amplification.

### 2.3. TFs with Moonlighting Functions at the Midbody

Many of the TFs that concentrate in the centrosomes are also enriched at the midbody. For example, ATF5, OCT1, and CTCF accumulate in the midbody dark zone, while SNAP45, ELK1, RUNX3, and Kaiso localize to regions that flank the dark zone (Table 1; Figure 1). The possible roles of these proteins in cytokinesis have not been thoroughly investigated. Thus, the reason for their double localization is intriguing but currently unknown. These proteins might play some direct functions in completion of cytokinesis, but it is also possible that they are hosted in the midbody without affecting cytokinesis. In this respect, it is worth noting that proteomic analysis of isolated midbodies has detected many proteins with no obvious roles in the cytokinetic process [87].

However, the core binding factor β (CBFβ/CBFB) and the YAP/YAP1 transcriptional regulator, appear to play moonlighting functions in cytokinesis. In human cells, CBFβ accumulates in the flanking regions of the midbody and co-precipitates with the RUNX TFs and the myosin regulatory light chain 3 (MRLC3/MYL12A) [88]. RNAi-mediated depletion of CBFβ or overexpression of the CBFβ-SMMHC (smooth muscle myosin heavy chain) leukemogenic fusion protein results in midbody abnormalities and mislocalization of PRC1 [88]. PRC1 cross-links antiparallel MTs in the midbody and is essential for completion of cytokinesis [89]. YAP is an interacting partner of the human RUNX proteins that accumulate in the midbody dark zone. YAP is phosphorylated during mitosis and this modification is essential for its function in cytokinesis. YAP co-precipitates with the polarity scaffold protein PATJ that is also enriched in the midbody dark zone and whose knockdown phenocopies the abscission defect elicited by YAP depletion [90]. Thus, although there is no compelling evidence for a role of the RUNX proteins in cytokinesis, the RUNX interacting partners CBFβ and YAP appear to have moonlighting functions in completion of cytokinesis.

### 2.4. TFs That Localize to the Spindle

Many TFs associate with interphase MTs and this association is thought to regulate TF ingress into the nucleus [91]. For example, it has been reported that p53 binds to MTs and is actively transported towards the nucleus in a dynein-dependent manner [92]. Other TFs exhibit a robust association with the spindle MTs, and some of them appear to have moonlighting mitotic functions. One of these factors is autoimmune regulator (Aire), a non-canonical TF involved in the immune tolerance processes [93]. Aire associates with the metaphase spindles and ana-telophase central spindles of mouse embryonic stem (mES) cells, and physically interacts with several spindle proteins including AURKB, the centrosomal proteins CEP55 and centrobin (CNTROB), the augmin-like proteins HAUS5 and HAUS8, and the CLASP1 and CLASP2 regulators of MT dynamics [94]. *Aire* conditional (floxed) knockout revealed that Aire loss results in morphologically abnormal spindle poles and centrosome amplification in mES cells [94]. These results strongly suggest that Aire has a moonlighting mitotic function but the mechanisms by which Aire controls spindle assembly and centrosome number remain to be determined.

Other transcriptional regulators with possible direct mitotic functions are the components of multiprotein complexes containing histone deacetylase 3 (HDAC3), which repress transcription [95]. HDAC3, the nuclear receptor corepressor (N-CoR/NCOR1), the transducin-β-like protein 1 (TBL1/TBL1X), and the TBL1-related protein 1 (TBLR1/TBL1XR1) all associate with the metaphase and anaphase spindles, but not with telophase spindles; RNAi-mediated depletion of either HDAC3 or N-CoR results in morphologically abnormal spindles and chromosome misalignment in HeLa cells [96]. It has been also shown that these mitotic defects are a specific consequence of disruption of HDAC3 deacetylase activity, whereas they are not elicited by treatment with the RNA polymerase II inhibitor α-amanitin [96]. Thus, even if there is no compelling evidence for a direct physical interaction of the HDAC3-associated proteins with spindle components, the extant data suggest that HDAC3 regulates mitotic spindle formation in a transcription-independent manner.

Two additional TFs with roles in spindle assembly are mouse Egr3 and TFIIB, which associate with the meiotic spindles of mouse females. Egr3 localizes to the spindles, but does not appear to interact with MTs, and its function in meiotic division has not been investigated [97]. TFIIB also localizes to meiotic spindles; RNAi against *TFIIB* or anti-TFIIB antibody injection into the oocyte results in morphologically abnormal spindles and chromosome misalignment. However, there are no data on biochemical interactions between TFIIB and spindle components [98]. Thus, current results do not allow a conclusion on whether Egr3 and TFIIB play direct roles in meiotic spindle assembly.

### 2.5. TFs with Roles in the Spindle Assembly Checkpoint (SAC)

TFs have been also implicated in the assembly and functioning of the SAC machinery. Several TFs have been shown to regulate transcription of the SAC components. As mentioned earlier, loss of RUNX1 negatively affects transcription of the *BUB1* and *NEK6* SAC genes [78]. Furthermore, *BRCA1* knockdown in human cancer cells downregulates transcription of multiple mitotic genes, including the *BUB1*, *BUBR1*/*BUB1B*, *AURKA*, *ESPL1*, and *PTTG1* SAC genes [99]. Similarly, *BRCA1* mutant mouse cells display decreased expression of *MAD2*/*MAD2L1*, *BUB1*, *BUBR1*, and *ZW10* [54,100]. Thus, besides its moonlighting role at centrosomes, BRCA1 also controls SAC by regulating transcription of its major components.

The WT1 TF and the TIF1γ/TRIM33 transcriptional intermediary factor are both functioning in the SAC-Anaphase Promoting Complex (APC/C) machinery, but while WT1 is likely to have a moonlighting function, TIF1γ does not fit all three criteria to be considered a moonlighting protein (Table 1). WT1 co-localizes and directly interacts with MAD2 throughout mitosis, and has high affinity for the active closed MAD2 conformation in human cells. RNAi-mediated knockout of *WT1* enhances turnover of the APC/C target securin (PTTG1), leading to an accelerated metaphase-to-anaphase transition and defects in chromosome segregation. Consistent with a direct mitotic role, WT1 loss does not significantly affect the expression of genes involved in SAC and APC/C regulation [101,102]. TIF1γ physically interacts with the APC/C and its CDC20 co-factor; *TIF1γ* knockdown in HeLa cells negatively regulates the E3 ubiquitin ligase activity of APC/C, causing inappropriate presence of cyclin A at metaphase, chromosome misalignment, and delay in anaphase onset [103,104]. Thus, WT1 and TIF1γ play opposite functions in the SAC-APC/C system, with WT1 delaying and TIF1γ accelerating metaphase-to-anaphase transition.

### 2.6. Mitotic and Meiotic Roles of the TFIIH Complex Components

TFIIH is an evolutionarily conserved multi-subunit complex required for transcription initiation. In humans, TFIIH consists of a seven-subunit core complex formed by XPB/ERCC3, p62/GTF2H1, p52/GTF2H4, p44/GTF2H2, p34/GTF2H3, p8/GTF2H5, and XPD/ERCC2, and a three-protein subcomplex including the CDK7 kinase, cyclin H (CCNH), and MAT1/MNAT1. Mutations in *XPB*, *XPD*, and *p8* lead to genetic diseases such as xeroderma pigmentosum, Cockayne syndrome, and trichothiodystrophy. XPD and XPB are not only involved in transcription, but also play direct functions in nucleotide excision repair (NER) [105,106,107].

Certain TFIIH subunits play roles in cell division. The first indication of a role of XPB in spindle formation came from studies on the *haywire* (*hay*) gene, which encodes the *Drosophila* ortholog of XPB [108]. A specific mutant allele of *hay* fails to complement mutations in the testis-specific *β2-tubulin* gene, which maps to a separate locus. In addition, males homozygous for this allele display defects in meiotic spindles, suggesting a role of *hay* in spindle assembly [108,109,110]. XPB has been also implicated in mammalian cell mitosis. XPB localizes to the centrosomes in both monkey and human cells and co-immunoprecipitates with γ-tubulin, but the functional significance of this localization has not been investigated [111].

Another TFIIH subunit with a potential mitotic role is XPD. XPD is also part of a five-protein complex independent of TFIIH that includes MMS19, MIP18/CIAO2B, CIAO1, and ANT2/SLC25A5. XPD, MMS19, and MIP18 localize to the mitotic spindle of human cells and RNAi-mediated depletion of each of these proteins results in monopolar and multipolar spindles, and in multinucleated cells [112]. Notably, fibroblasts derived from patients with certain *XPD* mutations show the same mitotic phenotype observed in HeLa *XPD* RNAi cells [112]. However, these results do not exclude the possibility that XPD deficiency might result in subtle defects in transcription leading to abnormal mitotic division [112].

The mitotic roles of the TFIIH subunits have been also analyzed in the *Drosophila* embryo system. Studies using fluorescently tagged TFIIH components have shown that Hay, p8, p52, and Cdk7 exhibit similar localization patterns in live early embryos. These proteins are enriched around the metaphase chromosomes and in some cases (e.g., p52) they appear to concentrate in a region corresponding to the spindle [113]. Moreover, in embryos from *p8* and *hay* mutant mothers or *Cdk7* RNAi mothers, most spindles are morphologically aberrant and show abnormal chromosome segregation [113]. *Drosophila* Xpd is required for proper spindle dynamics and chromosome segregation in early embryos. It has been suggested that loss of Xpd results in faulty localization of Cdk7, which is thought to cause mitotic defects [114].

In summary, while there are several indications that some TFIIH components might have direct mitotic roles in both humans and *Drosophila*, a solid proof is still lacking. Perhaps, the most compelling evidence for a moonlighting function of a TFIIH subunit is provided by the studies on the *Drosophila hay* (*XPB*) gene, which genetically interacts with the *β2-tubulin* gene and functions in meiotic spindle formation in males.

### 2.7. Mitotic Functions of the KANSL and NSL Complexes

The nonspecific lethal (NSL) complex is a highly conserved multiprotein assembly that regulates gene transcription in both *Drosophila* and mammals. The human NSL complex, also called KAT8-associated nonspecific lethal (KANSL), comprises 8 proteins (KANSL1, KANSL2, KANSL3, MCRS1, PHF20, WDR5, HCF1/HCFC1, and OGT) that associate with the MOF/KAT8 acetyltransferase to regulate transcription of a specific set of genes and contribute to stem cell identity [115,116,117]. The *Drosophila* NSL complex contains at least six evolutionarily conserved Mof-associated proteins, namely, Nsl1 (KANSL1), Dgt1 (KANSL2), Rcd1 (KANSL3), Rcd5 (MCRS1), MBD-R2 (PHF20), and Wds (WDR5). The *Drosophila* genome also harbors the *HCF1* and *OGT* orthologs, but it is currently unknown whether the protein products of these genes are complexed with the NSL subunits [117]. The *Drosophila* NSL complex acts as a major transcriptional regulator; its subunits bind to the promoters of more than 4000 housekeeping genes, although they appear to regulate transcription of only a subset of these genes [118,119].

The components of the human and *Drosophila* NSL complexes appear to have direct and indirect mitotic functions, providing insight into how transcription factors evolved moonlighting mitotic functions. KANSL1 and KANSL3 localize to the spindles of HeLa cells and are particularly enriched at the spindle poles. Consistent with this localization, KANSL3, and to a lesser extent KANSL1, bind the MT minus ends in vitro; MCRS1 does not bind the MT minus ends and is recruited to the spindle poles through interactions with KANSL3 and KANSL1 [120,121]. Furthermore, these three proteins physically interact with the TPX2 and MCAK/KIF2C spindle assembly factors. Depletion of each of the three proteins destabilizes the kinetochore fibers (the MT bundles that extend from the kinetochores to the spindle poles), resulting in chromosome misalignment and faulty segregation [120,121]. These data strongly suggest that KANSL1, KANSL3, and MCRS1 form a complex that associates with the spindle MT minus ends and directly controls spindle assembly and functioning (Table 1).

Besides being present in the KANSL complex, WDR5 also associates with the MLL_N_ and MLL_C_ subunits of the proteolytically cleaved mixed-lineage leukemia 1 (MLL1/KMT2A) protein, forming a complex with methyltransferase activity that catalyzes trimethylation of histone H3 lysine 4 (H3K4) and positively regulates gene expression [122,123]. The two MLL1 subunits and WDR5 localize to the centrosomes, the spindle, the central spindle, and the midbody dark zone in human cells [124,125,126] (Figure 1). WDR5 also interacts with the MT depolymerase KIF2A, promoting its localization to the spindle poles [125], and with several midbody proteins required for cytokinesis, including PRC1, CEP55, and the centralspindlin components MKLP1/KIF23 and CYK4/RACGAP1 [124]. Consistent with its localization and biochemical interactions, RNAi-mediated depletion of WDR5 results in morphologically abnormal and elongated spindles, aberrant chromosome behavior and segregation, and failures in cytokinesis [124,125]. Collectively, these results indicate a moonlighting mitotic role for WDR5, but this role does not appear to be the same as that played by KANSL1, KANSL3, and MCRS1.

The *Drosophila* components of the NSL complex are also implicated in cell division, but they do not appear to serve major moonlighting functions during mitosis. Genome-wide RNAi screens performed in S2 cells showed that Dgt1 (KANSL2) is required for γ-tubulin recruitment to centrosomes [17], while Rcd1 (KANSL3) and Rcd5 (MCRS1) are required for centriole duplication, and both centriole duplication and PCM recruitment at the centrosomes, respectively [18]. Another RNAi-based screen in S2 cells showed that both Rcd1 and MBD-R2 (PHF20) are required for mitotic chromosome segregation [19]. However, these studies did not determine the mitotic localization of Dgt1, Rcd1, Rcd5, and MBD-R2, nor addressed the mechanisms leading to the mitotic phenotypes elicited by depletion of these proteins.

These issues have been addressed in a more recent study that analyzed in greater detail the mitotic functions of Rcd1, Rcd5, MBD-R2, and Wds (WDR5) [127]. Expression of GFP-tagged forms of these proteins in S2 cells revealed that they are all restricted to the nucleus during interphase, but relocate to specific mitotic structures during mitosis. Both Rcd1-GFP and Rcd5-GFP accumulate at the centrosomes and the midbody and are excluded from the chromosomes, but while Rcd5-GFP accumulates in the midbody dark zone, Rcd1-GFP is enriched in the central spindles and in midbody flanking regions. In contrast, MBD-R2-GFP is exclusively enriched at the mitotic chromosomes, while Wds-GFP exhibits multiple localizations, namely, the centrosomes, the kinetochores of metaphase chromosomes, the midbody dark zone, and a discrete region of a specific chromosome [127] (Figure 1). Despite their different localization patterns, RNAi-mediated depletion of Rcd1, Rcd5, MBD-R2, or Wds results in identical mitotic defects, although the frequency of these defects is lower in Wds-deficient cells. Cells depleted of each of these proteins show fewer centrosomes than control cells, suggesting a defect in centriole duplication. In addition, the same cells display frequent failures in chromosome alignment and segregation. This latter phenotype has been attributed to reduction of the levels of both Cid (CENP-A) and Ndc80 [127]. Cid is a centromere-specific histone variant required for kinetochore assembly [128] and Ndc80 is the subunit of the kinetochore complex that binds to the spindle MTs [129]. Consistent with these phenotypes, transcription of many genes encoding centromere/kinetochore proteins (e.g., *cid*, *Mis12*, and *Nnf1b*), or involved in centriole duplication (e.g., *Sas-6*, *Sas-4*, and *asl*) is strongly reduced in *Rcd1*, *Rcd5*, and *MBD-R2* RNAi cells, and to a lesser extent in *wds* RNAi cells [127]. These results suggest that the mitotic functions of Rcd1, Rcd5, MBD-R2, and Wds are mostly indirect, with these proteins working together in interphase to stimulate transcription of genes required for centrosome duplication and kinetochore assembly (Table 1).

In summary, the studies on the human NSL/KANSL complex revealed that four components of this complex, KANSL1, KANSL3, MCRS1, and WDR5, meet our criteria to be genuine moonlighting proteins during mitosis; namely, they localize to specific mitotic structures, they biochemically interact with proteins enriched in these structures, and their depletion leads to defects in the same structures. However, the studies on human cells did not determine whether KANSL1, KANSL3, MCRS1, or WDR5 depletion leads to reduced transcription of critical mitotic genes, and it cannot therefore be excluded that these proteins could also have some indirect transcription-related mitotic roles. Conversely, the Rcd1, Rcd5, MBD-R2, and Wds components of the *Drosophila* NSL complex do not fit our criteria to be regarded as moonlighting proteins with mitotic functions, and instead appear to control cell division primarily through the transcriptional regulation of mitotic genes. However, it cannot be excluded that these NSL subunits have some minor direct mitotic functions that were not detected because they are redundant or phenotypically masked by the strong defects caused by reduced transcription of critical mitotic genes (Table 1).

## 3. Mitotic Functions of Splicing Factors (SFs)

RNAi-based genome-wide screens carried out both in *Drosophila* and human cells have identified many different SFs that are required for mitotic division [15,16,17,19,20] (Table S2). However, only a few of these SFs have been sufficiently characterized to determine whether they have a direct mitotic role or are instead controlling the splicing of mitotic pre-mRNAs (Table 2).

### 3.1. SFs with Indirect Mitotic Functions

An evolutionarily conserved SF that affects mitosis indirectly is encoded by the *S. cerevisiae cef1* gene. In *cef1* mutants, the single intron of the *α-tubulin* gene is not properly removed, resulting in reduction of the tubulin level and disruption of spindle assembly. Cells containing an intronless *α-tubulin* gene are resistant to *cef1* mutations, confirming that the splicing defect is responsible for the phenotype [130]. An indirect role of SFs in mitotic division is also suggested by studies on human U2AF35/U2AF1. HeLa cells treated with *U2AF35* siRNAs exhibit strong defects in spindle morphology and chromosome segregation; these defects have been attributed to defective splicing of the pre-mRNA encoding the CDC27 component of the APC/C complex. The localization of U2AF35 during mitosis has not been investigated [131].

SON is another SF that does not appear to localize to mitotic structures; its depletion in HeLa cells causes failures of centrosome migration to the opposite cell poles during prophase, and defective chromosome alignment and segregation. These defects were ascribed to inadequate RNA splicing of a specific set of genes, including those encoding *ɣ*-tubulin and the *ɣ*TuRC component TUBGCP2 [132]. WBP11 and NRDE2 are two additional SFs that indirectly control centrosome behavior. WBP11 depletion in human cells results in the retention of multiple introns in the *TUBGCP6* mRNA that specifies another *ɣ*TuRC component, leading to defective centriole duplication. This defect is partially rescued by an intronless *TUBGCP6* transgene, supporting the idea that WBP11 controls centriole behavior indirectly [133]. NRDE2 is a human SF whose depletion results in defective splicing of the pre-mRNA encoding the centrosomal protein CEP131. Reduction of the CEP131 level impairs recruitment of proteins such as AURKA, γ-tubulin, and CEP192 to the centrosomes, and leads to defective chromosome segregation. During mitosis, NRDE2 is diffuse in the cytoplasm and fails to accumulate in any mitotic structure [134].

### 3.2. SFs That Control Cohesin Behavior

In 2014, work from four different research groups led to the identification of 27 SFs that control sister chromatid cohesion in human cells [135,136,137,138]. The multi-subunit cohesin complex holds the sister chromatids together until anaphase, ensuring a faithful chromosome segregation. Cohesin associates with DNA following replication, and this association is stabilized by sororin/CDCA5 that antagonizes WAPL, a protein that is able to release cohesin from DNA, thereby allowing sister chromatid separation [139]. Seven of the 27 SFs affecting cohesin behavior have been analyzed in detail: NHP2L1/SNU13, SART1, MFAP1, CDC5L, SNW1, PRP19/PRPF19, and UBL5. RNAi-mediated depletion of each of these SFs in human cells resulted in metaphase chromosomes with parallel sister chromatids, anaphase delay, and defective chromosome segregation. Transcript analysis revealed that depletion of these factors results in intron retention in the *sororin* mRNA, but not in mRNAs encoding cohesin subunits. Accordingly, the SF-dependent mitotic defect was partially restored by either the expression of an intronless *sororin* transgene or RNAi against *WAPL.* These results suggest that many SFs mediate sister chromatid cohesion indirectly, predominantly by catalyzing splicing of the *sororin* pre-mRNA [135,136,137,138].

However, since the phenotypic rescue elicited by intronless *sororin* expression is often incomplete, SFs might contribute to sister chromatid cohesion in some sororin-independent way. One possibility is that SFs directly regulate cohesin association with the chromosomes, as suggested by recent work showing that many SFs physically interact with cohesin [140]. Each of the 11 known components of cohesin was tagged with a dual FLAG-SBP peptide and expressed in cultured human cells; dual affinity purification followed by mass spectrometry revealed that the cohesin subunits copurify with a large number of SFs, including some SFs that were previously shown to be required for sister chromatid cohesion [136]. Furthermore, it has been demonstrated that in human cells, the cohesin subunit STAG2 and some of its interacting SFs (HNRNPH/HNRNPH1, EFTUD2, and SF3B1) precisely colocalize throughout the cell cycle [140]. In conclusion, while it is likely that several SFs control cohesin behavior primarily through the regulation of *sororin* pre-mRNA splicing, the robust physical interaction between cohesin and SFs raises the concrete possibility that some SFs contribute directly to sister chromatid cohesion.

### 3.3. The Indirect and Direct Mitotic Functions of the PRP19 Complex

The PRP19 complex (also known as nineteen complex or NTC) is a multifunctional protein assembly that has a conserved core of four subunits (PRP19/PRPF19, CDC5L, SPF27/BCAS2, and PLRG1). Besides splicing, this complex has been implicated in several additional processes, including DNA damage response and protein ubiquitination [141,142]. The PRP19 complex is also involved in mitosis, where it appears to play both indirect and direct functions. RNAi-mediated depletion of each component of the complex in human cells impairs kinetochore-MT attachment, causing frequent failures in chromosome segregation [143]. These mitotic aberrations are attributed to defective splicing of mitotic genes such as those that encode the dynein-dynactin components DYNC1H1, DYNLRB2, and DCTN4, which are required for proper kinetochore-microtubule attachment [143].

As already mentioned, other studies showed that PRP19- and CDC5L-depleted human cells exhibit reduced cohesion between sister chromatids, and that this defect is rescued by the expression of an intronless *sororin* gene [136,138]. However, the same cells, in addition to the cohesion defect, display morphologically abnormal mitotic spindles. These aberrant spindles are still present in cells expressing intronless *sororin* and are not observed in cells depleted of cohesin subunits [138], suggesting a cohesin-independent requirement of the PRP19 complex for spindle assembly.

There is an additional study that points to a splicing-independent, direct mitotic role of the PRP19 complex [42]. siRNA-mediated knockdown of *SPF27* in HeLa cells leads to downregulation of the other components of the PRP19 complex and to a severe defect in end-on attachment of MTs to the kinetochores [42], consistent with the results of Mu and coauthors (2014) [143]. Moreover, an investigation of the role of the PRP19 complex in spindle assembly around sperm nuclei in *Xenopus* egg extracts suggests a direct mitotic function. *Xenopus* extracts immunodepleted of either the PRP19 or the SPF27 ortholog produce spindles with reduced MT density in the central part, suggesting a defect in the interaction between the sperm chromatin and the spindle MTs [42]. These results argue strongly for a moonlighting, splicing-independent mitotic function of the PRP19 complex because, as mentioned earlier, *Xenopus* egg extracts can form a spindle in the complete absence of transcription or translation of any mRNA, except *cyclin B*.

Collectively, these results suggest a dual mitotic function for the PRP19 complex, an indirect function required for splicing of pre-mRNAs encoding sororin and some dynein-dynactin components and a direct, as yet undefined function in mitotic spindle assembly.

### 3.4. SFs with Roles in the SAC and in Kinetochore-MT Interactions

A protein involved in pre-mRNA splicing that is also required for SAC activity in human cells is the PRP4/PRPF4 kinase. PRP4 is required for pre-mRNA splicing in *S. pombe* [144] and tightly associates with U4/U6 snRNP in human cells [145,146]. PRP4 accumulates on the kinetochores of HeLa cells and mediates recruitment of SAC proteins such as MPS1/IDUA, MAD1/MAD1L1, and MAD2 to kinetochores, but there is no evidence of physical interaction between PRP4 and these SAC components [147]. PRP4 depletion results in precocious anaphase onset, lagging anaphase chromosomes, and aneuploidy, indicating that PRP4 deficiency compromises SAC activity [147]. Thus, PRP4 does not fit all three criteria to be considered a moonlighting protein. However, in PRP4-depleted cells, the biogenesis of SAC proteins such as BUBR1/BUB1B, MAD1, MAD2, and MPS1 is not affected, suggesting a direct role of PRP4 in the SAC machinery [147].

Studies in *Drosophila* and human cells have recently suggested that the conserved spliceosomal components SF3A2 and PRP31 (designated as SF3A2 and PRP31/PRPF31 in humans, and Sf3A2 and Prp31 in *Drosophila*) play direct mitotic roles. SF3A2 is a subunit of a conserved heterotrimeric SF3A complex of U2 snRNP; PRP31 is a component of the U4/U6.U5 tri-snRNP complex that comprises at least 30 proteins [148,149,150]. *Drosophila* Sf3A2 and Prp31 associate with the spindles of S2 tissue culture cells and bind MTs in vitro [151]. The mouse ortholog of Sf3A2 was also shown to have MT binding and bundling activities [152], suggesting that Sf3A2 plays a conserved non-canonical MT-binding function. Furthermore, Sf3A2 and Prp31 physically interact with each other and directly bind the components of the *Drosophila* Ndc80 complex (Ndc80, Mitch/Spc25, and Nuf2). Consistent with these findings, depletion of these SFs in *Drosophila* cells impairs accumulation of Ndc80 at kinetochores, leading to severe defects in chromosome alignment and segregation [151]. Comparable results were obtained in HeLa cells where SF3A2 and PRP31 were found to interact physically with each other and with HEC1/NDC80. In addition, experiments with synchronized cells showed that the SF3A2-HEC1 and PRP31-HEC1 interactions are restricted to the M phase of the cell cycle. Loss of either SF in HeLa cells results in morphologically irregular spindles, defective chromosome congression at metaphase, and reduced frequency of ana-telophases [151]. Collectively, these results suggest that SF3A2 and PRP31 directly regulate interactions among kinetochores, spindle MTs, and the Ndc80 complex in both *Drosophila* and human cells.

Further strong evidence for a direct mitotic role of Sf3A2 and Prp31 is provided by the results of antibody injections into *Drosophila* embryos [151]. After fertilization, *Drosophila* syncytial embryos undergo 13 extremely rapid nuclear divisions that predominantly rely on already spliced mRNAs and proteins packaged into the egg by the mother, although a minor transcription wave has been reported during cycles 8–13 [153,154]. These early transcripts require the Fandango subunit of the Prp19 complex for splicing, but loss of Fandango does not affect syncytial mitoses, causing nuclear defects only during the 14th cell cycle [155]. Thus, acute inactivation of a SF by antibody injection in live early embryos (cycles 8–13) is not expected to cause splicing-dependent mitotic defects, but only defects reflecting a direct mitotic role of the targeted SF. Syncytial embryos injected with either anti-Sf3A2 or anti-Prp31 antibodies shortly before nuclear envelope breakdown (NEB) display very similar mitotic phenotypes. Mitotic divisions show a decrease in spindle length relative to controls, defective chromosome alignment at metaphase, and frequent failures in chromosome segregation. This phenotype is observed as early as one minute after antibody injection, ruling out the possibility of an indirect mitotic effect of Sf3A2 and Prp31 [151]. Indeed, one minute does not appear to be sufficient for splicing and translation of a hypothetical mitotic pre-mRNA and, most importantly, it is extremely unlikely that the protein product of this pre-mRNA needs to be replenished every minute (Table 2).

## 4. Conclusions and Perspectives

The studies described here implicate many transcription and splicing factors in the regulation and proper execution of mitotic division. Several of these factors do not localize to specific mitotic structures and appear to affect mitosis indirectly by controlling transcription or splicing of pre-mRNAs that encode mitotic proteins. Other factors are enriched in specific mitotic structures, but the extant data are not sufficient to establish whether they play direct mitotic functions. A third category of factors localizes to a specific mitotic structure(s), biochemically interacts with proteins enriched in same structure(s), and causes defects in this/these structure(s) upon depletion. We have assumed that the TFs and SFs with these three properties play moonlighting mitotic functions besides their canonical functions in RNA transcription or splicing. We believe that these three properties are sufficient to conclude that a TF or a SF directly functions in mitosis. However, this conclusion would be consolidated by the demonstration that depletion of the factor under study leads to mitotic defects in a model system where mitosis is both transcription- and splicing-independent. As mentioned earlier, examples of these systems are the *Xenopus* egg extracts immunodepleted of a specific factor [39,41,42] and live early *Drosophila* embryos expressing suitable fluorescent mitotic markers injected with specific antibodies. The latter system has the additional advantage of permitting the analysis of a large number of synchronous mitoses within minutes from the antibody injection [151].

The extant data indicate that the moonlighting TFs and SFs are involved in diverse mitotic processes, including (i) centriole stability, duplication, and PCM recruiting ability; (ii) centrosome- and chromatin-driven MT nucleation; (iii) spindle formation and dynamics; (iv) kinetochore-MT interaction; (v) spindle assembly checkpoint; and (vi) cytokinesis. Thus, it appears that most if not all steps of mitosis are directly regulated by some TFs and/or SFs. This raises the question of how TFs and SFs evolved direct mitotic functions. The evolution and the biological meaning of protein moonlighting are discussed in detail in the review of Copley (2014) [4] and in the recent book by Henderson et al. (2017) [5]. Here, we only consider the simplest scenario, assuming than TFs and SFs gradually evolved so as to acquire an additional mitotic function, while keeping their canonical roles in transcription or splicing. It is likely that the TFs and SFs described here evolved a moonlighting mitotic function through the acquisition of genetic changes that allow physical interactions with mitotic proteins, specific posttranslational modifications, or both (Table 1 and Table 2; Figure 1). In this context, it is worth noting that published work (see Table 1 and Table 2) indicates that the TFs with putative moonlighting mitotic functions outnumber the SFs with the same functions. These data raise the question of whether TFs are more prone than SFs to evolve additional mitotic functions. This is an unlikely possibility for at least two reasons. First, the global number of TFs is at least five times higher than that of SFs [21,156,157]. Second, and most importantly, a simple search of the PubMed database using “transcription factors” as query retrieved 39 times more publications than those retrieved using “splicing factors”. Thus, taking into account that RNAi-based screens yielded more SFs than TFs (Tables S1 and S2), it is conceivable that future work will identify many additional SFs with moonlighting mitotic functions.

Many TFs accumulate in specific mitotic structures, but there is not compelling evidence that these accumulations are functionally relevant (Table 1; Figure 1). In addition, some conserved TFs appear to have species-specific mitotic localizations and functions. For example, the mitotic localizations of the human KANSL complex subunits are not identical to those of their NSL counterparts in *Drosophila*. In addition, while the KANSL components appear to have integral mitotic roles, their NSL orthologs are primarily involved in transcriptional regulation of specific mitotic genes [120,121,127]. However, it has been suggested that the *Drosophila* NSL proteins might have relatively minor direct mitotic roles that are masked by their transcription-dependent effects on cell division [127]. More in general, we propose that most proteins that accumulate in mitotic structures have either minor moonlighting functions or are evolving towards the acquisition of these functions. Finally, we would like to note that the TFs and SFs with established or possible moonlighting mitotic functions ensure the fidelity of a series of processes, whose alteration can promote carcinogenesis, namely, centrosome duplication, kinetochore-MT interaction, spindle assembly checkpoint, and cytokinesis. Many of these factors have been already implicated in cancer promotion or suppression (Table 1 and Table 2) and their further characterization is likely to provide additional insight into their roles in tumor development. We also believe that identification of new TFs and SFs with moonlighting mitotic functions should be an important goal of future studies, as a detailed understanding of the multiple mechanisms that regulate mitotic division will be instrumental to devise new cancer therapies.

## Figures and Tables

**Table 1:** Mitotic roles of transcription factors.

Name (Organism)	Mitotic Localization	Interacting Mitotic Proteins	Loss-of-Function Mitotic Phenotype	Mitotic Function (Role in Cancer)
Sak1, Fkh2 (Sp)	ND	ND	Regulate mitotic gene transcription. Defective septation [24].	Indirect
FKHRL1/FOXO3 (h)	ND	ND	Regulates mitotic gene transcription. Defective mitotic exit and cytokinesis [23].	Indirect (TS)
Zas1 (Sp)	Chromosomes	ND	Regulates mitotic gene transcription. Defective chromosome condensation and segregation [25].	Indirect
ERG (h)	RNA processing bodies (PBs)	ND	Degrades *AURKA* and *AURKB* mRNAs. Multipolar spindles and aberrant spindle structure [26].	Indirect (TP)
BRCA1 (h)	Not relevant	Not relevant	Regulates transcription of multiple mitotic genes, including *BUB1*, *BUBR1/BUB1B*, *AURKA*, *ESPL1*, *PTTG1*, *ASPM*, *PRC1*, and *PLK1* [99].	Indirect (TS)
BRCA1 (m)	Not relevant	Not relevant	Regulates SAC gene transcription, including *MAD2*, *BUB1*, *BUBR1*, and *ZW10* [54,100].	Indirect (TS)
BRCA1 (h)	Centrosomes	γ-tubulin	Centrosome amplification and fragmentation [31,32,33,34,35,36,37].	DIRECT (TS)
BRCA1 (Xe)	Spindle poles	TPX2, NuMa, XRHAMM	Morphologically abnormal spindles in BRCA1-immunodepleted *Xenopus* egg extracts [40].	DIRECT
ATF5 (h)	Centrosomes (mother centriole) Midbody DZ	γ-tubulin, PCNT	Defective accumulation of PCM; centriolar fragmentation, multipolar spindles; unknown function at the midbody [44,45].	DIRECT
AKNA (m)	Centrosomes (mother centriole)	γTuRC, EB1, DCTN1	Defective centrosome-driven MT regrowth after MT depolymerization [49].	DIRECT
YB-1/YBX1 (h)	Centrosomes Spindle poles	γ-tubulin, PCNT	Structurally abnormal centrosomes with reduced MT nucleation ability [50,51].	DIRECT (TP)
OCT1/POU2F1 (h) Interacts with BRCA1	Centrosomes Kinetochores Midbody DZ	PARP-1, APC1	Abnormal mitosis; unknown function at the midbody [52,53,54,55].	DIRECT (TP)
Oct1 (Xe)	ND	ND	Morphologically abnormal spindles in Oct1-immunodepleted *Xenopus* egg extracts [52].	DIRECT
Nubbin (Dm) (OCT1 ortholog)	Central spindle Midbody FR	ND	Unknown function at the midbody [56].	P-direct ND
p53/TP53 (h)	Centrosomes	ND	Centrosome amplification and fragmentation [60,61,65,66,67].	P-direct (TS)
p53 (m)	Centrosomes	ND	Centrosome amplification [58,62,63,64].	P-direct
SP1 (h)	Centrosomes	ND	Centrosome amplification and decreased centrosome-driven MT nucleation [68].	P-direct (TP)
SP1 (m)	Centrosomes	ND	Centrosome amplification and decreased centrosome-driven MT nucleation [68].	P-direct
SF-1/NR5A1 (m)	Centrosomes	ND	Centrosome amplification [69,70].	P-direct
Kaiso/ZBTB33 (h)	Centrosomes Spindle Central spindle Midbody FR	γ-tubulin, PCNT	No obvious mitotic defects [71,72].	P-direct MR (TS)
CTCF (h) Interacts with Kaiso	Centrosomes Spindle poles Midbody DZ	ND	Mitotic phenotype not investigated [73,74].	P-direct ND (TS)
SNAP45/SNAPC2 (h)	Centrosomes Spindle poles Midbody FR	ND	Multiple mitotic defects; defective chromosome condensation [75].	P-direct
ELK1 (h)	Centrosomes Spindle Central spindle Midbody FR	AURKA	No obvious mitotic defects [20,76].	P-direct MR (TP, TS)
RUNX1 (h)	Not relevant	Not relevant	Regulates transcription of *BUBR1*, *BUB1*, and *NEK6* [78].	Indirect (TP)
RUNX1, RUNX2, RUNX3 (h)	Centrosomes Spindle Midbody FR	γ-tubulin, rootletin	Reduced cyclin B1 accumulation and delayed mitotic entry. No specific mitotic defects [79,80].	P-direct MR (TP)
CBFβ/CBFB (h) Binds RUNX proteins	Midbody FR	MRLC3	Defective midbody structure and cytokinesis (abscission); polyploid cells [88].	DIRECT (TS, TP)
YAP/YAP1 (h) Binds RUNX proteins	Midbody DZ	PATJ	Defective cytokinesis (abscission) [90].	DIRECT (TS, TP)
Aire (m) (AIRE in humans)	Spindle Central spindle	AURKB, CEP55, CNTROB, HAUS5, HAUS8, CLASP1, CLASP2	Abnormal spindle poles and centrosome amplification [94].	DIRECT
HDAC3 (h) (in complex with NCOR1, TBL1X and TBL1XR1)	Spindle	ND	Morphologically abnormal spindles and chromosome misalignment [96].	P-direct (TP)
Egr3 (m)	Meiotic spindles of mouse females	ND	Meiotic phenotype not investigated [97].	P-direct ND
TFIIB (m) (GTF2B in humans)	Meiotic spindles of mouse females	ND	Morphologically abnormal spindles and chromosomes misalignment after antibody injection [98].	P-direct
WT1 (h)	Kinetochores (colocalizes with MAD2)	MAD2	Accelerated metaphase-to-anaphase transition; defective chromosome segregation [101,102].	DIRECT (TS, TP)
TIF1γ/TRIM33 (h)	ND	CDC20, APC/C	Chromosome misalignment; delay in metaphase-to-anaphase transition [103,104].	P-direct (TS, TP)
XPB/ERCC3 (h) (TFIIH subunit)	Centrosomes Spindle poles	γ-tubulin	Mitotic phenotype not investigated [111].	Direct ND (TS)
Hay (Dm) (TFIIH subunit, orthologous to XPB)	Chromosome/ spindle area in embryos	Testis-specific β2-tubulin	Defective meiotic spindles in males; abnormal mitotic spindles and defective chromosome segregation in embryos from *hay* mutant mothers [108,109,110,113].	P-direct
p8, p52, Cdk7 (Dm) (TFIIH subunits)	Chromosome/ spindle area in embryos	ND	Abnormal mitotic spindles and defective chromosome segregation in embryos from mutant or RNAi mothers [113].	P-direct
XPD/ERCC2 (h) (TFIIH subunit forms another complex with MIP18 and MMS19)	Spindle (with MIP18 and MMS19)	ND	Multipolar spindles and multinucleated cells [112].	P-direct
Xpd (Dm) (TFIIH subunit)	ND	Regulates Cdk7 localization	Abnormal mitotic spindles and defective chromosome segregation in embryos from *Xpd* mutant mothers [114].	P-direct
KANSL1, KANSL3, MCRS1 (h) (KANSL complex subunits)	Spindle poles; KANSL1 and KANSL3 bind MT minus ends	TPX2, MCAK	Chromosome misalignment and defective segregation [120,121].	DIRECT (TP)
WDR5 (h) (KANSL subunit that forms another complex with MLL1/KMT2A)	Centrosomes Spindle Central spindle Midbody DZ	KIF2A, PRC1, MKLP1, CYK4, CEP55	Morphologically abnormal and elongated spindles, aberrant chromosome segregation, and failures in cytokinesis [124,125,126].	DIRECT (TP)
Dgt1 (Dm) (NSL subunit orthologous to KANSL2)	Diffuse (weak on spindle)	ND	Diminished γ-tubulin at centrosomes; long spindles [17].	P-direct
Rcd1 (Dm) (NSL subunit orthologous to KANSL3)	Centrosomes Central spindle Midbody FR	ND	Defective centriole replication; frequent failures in chromosome alignment and segregation [18,19,127].	Indirect (P-direct MR)
Rcd5 (Dm) (NSL subunit orthologous to MCRS1)	Centrosomes Midbody DZ	ND	Defective centriole replication; frequent failures in chromosome alignment and segregation [17,18,127].	Indirect (P-direct MR)
MBD-R2 (Dm) (NSL subunit orthologous to PHF20)	Chromosomes	ND	Defective centriole replication; frequent failures in chromosome alignment and segregation [19,127].	Indirect (P-direct MR)
Wds (Dm) (NSL subunit orthologous to WDR5)	Centrosomes Kinetochores Midbody DZ	ND	Defective centriole replication; frequent failures in chromosome alignment and segregation [127].	Indirect (P-direct MR)

**Name:** Each human TF is designated either with its HUGO acronym only, or both the name used in the cited papers and the HUGO acronym. Organism: h, human; m, mouse; Dm, *Drosophila melanogaster*, Sp., *Schizosaccharomyces pombe*, Xe, *Xenopus* egg extracts. Mitotic localization: ND, not determined; Midbody DZ, midbody dark zone; Midbody FR, midbody flanking regions; localization to specific midbody regions has been decided based on the examination of published microphotographs. Interacting mitotic proteins: ND, not determined. Mitotic function: Indirect, controls transcription of mitotic genes; DIRECT, in addition to a role in transcription has a direct role in mitosis, namely it is a moonlighting protein that meets the 3 criteria specified in the text. P-direct, putatively direct, does not meet the 3 criteria but (in the cited papers) is thought to have a direct mitotic role. P-direct ND, localizes to a mitotic structure but its mitotic role has not been investigated. P-direct MR, localizes to a mitotic structure but its loss does not result in a detectable mitotic phenotype; it might therefore have either a minor or a redundant mitotic function. Role in cancer was determined by examination of the pertinent literature; references are not reported: TS, tumor suppressor; TP, tumor promoter; TS, TP, can function either as a tumor suppressor or promoter, depending on the cellular context.

**Table 2:** Mitotic roles of splicing factors.

Name (Organism)	Mitotic Localization	Interacting Mitotic Proteins	Loss-of-Function Mitotic Phenotype	Mitotic Function (Role in Cancer)
CEF1 (Sc)	ND	ND	Inefficient splicing of *α-tubulin* pre-mRNA. Defective spindle assembly. Mutant phenotype rescued by an intronless *α-tubulin* gene [130].	Indirect
U2F35/U2AF1 (h)	ND	ND	Inefficient splicing of the *CDC27* pre-mRNA. Defective spindle morphology and chromosome segregation [131].	Indirect (TS, TP)
SON (h)	ND	ND	Inefficient splicing of *TUBG1*, *TUBGCP2*, *TUBGCP4*, and *AKT1* pre-mRNAs. Impaired centrosome separation at prophase; defective chromosome segregation and cytokinesis [132].	Indirect (TS)
WBP11 (h)	ND	ND	Inefficient splicing of *TUBGCP6* pre-mRNA. Impaired centriole duplication; mutant phenotype rescued by an intronless *TUBGCP6* transgene [133].	Indirect
NRDE2 (h)	Diffuse in the cytoplasm	ND	Inefficient splicing of *CEP131* pre-mRNA. Impaired centrosomal recruitment of γ-tubulin, AURKA, and CEP192; defective chromosome segregation [134].	Indirect (TS, TP)
27 different human SFs, including PRPF8, NHP2L1/SNU13, SART1, MFAP1, CDC5L, SNW1, PRP19/PRPF19, UBL5, SF3B1, SNRNP200, PRPF6	Some colocalize with the Cohesin complex	Some copurify with the Cohesin subunits	Intron retention in the *sororin* mRNA. Parallel sister chromatids at metaphase and defective chromosome segregation; mutant phenotype partially rescued by an intronless *sororin* transgene [135,136,137,138,140].	Indirect, (possibly also DIRECT) (TP: CDC5L, SNW1, U2AF2)
PRP19/PRPF19 (h), CDC5L (h), SPF27/BCAS2 (h), PLRG1 (h) (PRP19 complex)	Not relevant	Not relevant	Inefficient splicing of *DYNC1H1*, *DYNLRB2*, and *DCTN4* pre-mRNAs. Impaired kinetochore-MT attachment, defective chromosome segregation [143].	Indirect (TP: CDC5L, BCAS2)
PRP19/PRPF19 (h), CDC5L (h), SPF27/BCAS2 (h) (PRP19 complex)	Diffuse	ND	Defective kinetochore-MT interaction; abnormal spindles and defective chromosome alignment in human cells [42,138].	P-direct (TP: CDC5L, BCAS2)
Prp19 (Xe), Bcas2 (Xe) (PRP19 complex)	ND	ND	Defective chromatin-MT interaction; morphologically abnormal spindles in *Xenopus* egg extracts [42].	DIRECT
PRP4/PRPF4 (h)	Kinetochores	ND	Defective recruitment at kinetochores of the MPS1, MAD1, and MAD2 SAC proteins. Precocious anaphase onset; lagging anaphase chromosomes and aneuploidy [147].	P-direct (TP)
SF3A2 (h), PRP31/PRPF31 (h)	Spindle (Mouse Sf3A2 binds MTs in vitro)	Ndc80/HEC1	Morphologically irregular spindles; defective chromosome congression at metaphase; reduced frequency of ana-telophases [151,152].	DIRECT
Sf3A2 (Dm), Prp31 (Dm)	Spindle (Binds MTs in vitro)	Ndc80, Mitch, Nuf2 (Ndc80 complex)	Reduced accumulation of Ndc80 at kinetochores and severe defects in chromosome alignment and segregation. Anti-Sf3A2 and anti-Prp31 injections disrupt mitosis in embryos [151].	DIRECT

**Name:** Each human SF is designated either with the HUGO acronym only, or both the name used in the cited papers and the HUGO acronym. Organism: h, human; m, mouse; Dm, *Drosophila melanogaster*; Sc, *Saccharomyces cerevisiae*; Xe, *Xenopus* egg extracts. Mitotic localization: ND, not determined. Interacting mitotic proteins: ND, not determined. Mitotic function: Indirect, control splicing of mitotic gene pre-mRNAs; DIRECT, in addition to a role in splicing has a direct role in mitosis, namely it is a moonlighting protein that meets the 3 criteria specified in the text. P-direct, putatively direct, does not meet the 3 criteria but (in the cited papers) is thought to have a direct mitotic role. Role in cancer was determined by examination of the pertinent literature; references are not reported: TS, tumor suppressor; TP, tumor promoter; TS, TP, can function either as a tumor suppressor or a tumor promoter, depending of the cellular context.

## Supplementary Materials

The following are available online at https://www.mdpi.com/2073-4409/9/6/1554/s1, **Table S1**: Transcription factors with mitotic functions identified in large-scale RNAi screens carried out in human and *Drosophila* tissue culture cells. **Table S2**: Splicing factors with mitotic functions identified in large-scale RNAi screens carried out in human and *Drosophila* tissue culture cells.
